# Rheopheresis in treatment of idiopathic sensorineural sudden hearing loss

**DOI:** 10.1186/s40463-017-0228-9

**Published:** 2017-06-29

**Authors:** Milan Kostal, Jakub Drsata, Milan Bláha, Miriam Lánská, Viktor Chrobok

**Affiliations:** 1 4th Department of Internal Medicine, University Hospital Hradec Kralove Charles University, Faculty of Medicine in Hradec Kralove, Hradec Králové, Czech Republic; 2 Department of Otorhinolaryngology and Head and Neck Surgery, University Hospital Hradec Kralove Charles University, Faculty of Medicine in Hradec Kralove, Hradec Králové, Czech Republic

**Keywords:** Rheopheresis, Sudden idiopathic hearing loss, MicroWick

## Abstract

**Backround:**

Only few therapeutic options exist for patients with refractory sudden idiopathic sensorineural hearing loss (SISHL). Little is known about the efficacy of second-line therapies. Rheopheresis seems to be an effective therapeutic possibility.

**Methods:**

Between 2012 and 2015, 106 patients with SISHL were enrolled in the study, of whom 52 were refractory to initial treatment. As salvage therapy, these patients were offered either 3 sessions of rheopheresis (33 pts) or intratympanic steroid treatment through MicroWick application (19 pts). Pure tone audiometry was performed at diagnosis, at the 1st month and the 1st year during the follow-up.

**Results:**

Patients in the rheopheretic arm had higher hearing loss than in the MicroWick arm (81% vs. 52%, *p* = 0.04). In spite of this, there was a significant improvement for patients in the rheopheretic arm (27% of hearing loss reduction, *p* < 0.001) after the 1st month and this remained unchanged during the 1st year, while no improvement was seen in the MicroWick arm (0% of hearing loss reduction, *p* = 0.424). We found no predictive factor for steroid-failure in first-line therapy. Older age (*p* = 0.003), presence of vertigo (*p* = 0.006) and more profound initial hearing loss (*p* < 0.001) were identified as negative prognostic markers.

**Conclusion:**

Rheopheresis can be used as a potentially effective and safe salvage therapy for patients with cortico-refractory SISHL.

## Background

Sudden hearing loss is defined as a hearing loss of over 30 dB with an acute onset (i.e. within a 72-h period), in at least 3 consecutive frequencies in one or both ears [1]. It has a global incidence rate of 5–20 cases in 100,000 people per year. This number may be underestimated because of diffuse diagnostic criteria and spontaneous remissions, which produces a case drop-off for statistical purposes [2]. Sudden hearing loss can be associated with specific causes, such as inflammatory, mechanic, chemical or acoustic damage of the cochlea, Ménière’s disease, vestibular schwannoma and others [3]. However about 90% of cases remain idiopathic [4]. Four major hypotheses have been proposed for these cases – traumatic, vascular, autoimmune and infectious [5]. The fact that the labyrinthine artery is a functional end artery (very vulnerable to vascular events) [6, 7] is in favor of the vascular hypothesis. The main underlying cause is atherosclerosis and its risk factors, such as abdominal obesity, arterial hypertension, hyperglycemia, hypertriglyceridemia, low HDL cholesterol or high BMI [8]. Other risk factors include thrombophilias and hyperviscosity syndrome [7].

The cochlear blood flow can be impaired by several synergistic factors causing vessel injury and elevating blood viscosity. Among them, a high level of cholesterol and fibrinogen (major factors of blood hyperviscosity) are a possible target for treatment with fibrinogen–LDL-apheresis, covering some of the currently established methods of lipidapheresis/rheopheresis [9, 10]. Rheopheresis simultaneously eliminates an exactly defined spectrum of high-molecular weight rheologically relevant plasma proteins (i.e. alpha-2-macroglobulin, fibrinogen, LDL cholesterol, von Willebrand factor (vWF), IgM, fibronectin, putatively multimeric vitronectin), thus lowering full blood and plasma viscosity [11]. These procedures have pleiotropic effects, including favorable modifications of cytokine and adhesive molecule levels, increased production of endothelial NO, improved erythrocyte deformability and reduced aggregability of both erythrocytes and platelets [7, 12]. Improvement of perfusion in inner ear microcirculation as a result of lower blood viscosity is a possible therapeutic approach in patients with SISHL.

## Study objective and methods

The primary objective of the study is to prove the efficacy of rheopheresis and MicroWick in patients with SISHL, for whom the first-line corticosteroid therapy has failed. We conducted an open-label observational prospective study with rheopheresis and MicroWick in steroid-refractory patients between 2012 and 2015 in a university-based tertiary care hospital. Institutional Review Board approval was obtained before proceeding with the study.

Patients meeting SISHL criteria (hearing loss of over 30 dB, in at least 3 consecutive frequencies in one or both ears within a 72-h period) were enrolled after signing informed consent. To exclude known causes of hearing loss, a complete history and physical examination, audiological and vestibular tests, laboratory workup and imaging study (MRI or CT, if indicated) were undertaken. All patients received corticosteroid therapy, consisting of 250 mg of solumedrol administered on 3 consecutive days (total of 750 mg of corticosteroid); then the assessment was performed (Days 3–5). Patients with a response of less than 50% improvement in PTA (pure tone average) were considered as partial or non-responders to the treatment [13]. These were offered a continuation of the therapy with MicroWick (intratympanic application of 7.5 mg dexamethasone in total) or rheopheresis. We collected demographic data (age, sex, time to treatment – TTT, BMI). A pure-tone audiogram and the Fowler percentage of hearing loss was performed before the treatment and during follow-up at the 1st and 12th months (final outcome) after treatment. PTA was calculated as the dB average of the thresholds at 0.5, 1, 2, and 4 kHz. The Fowler percentage of hearing loss was calculated according to the AMA (American medical association) standards, which has been proven as the optimal method for percentage evaluation of hearing loss in Czech language [14]. The presence of tinnitus or vertigo was noted. Adverse events were scored, using Common Terminology Criteria for Adverse Events (CTCAE v. 4.0, 7/2010).

We used rheohemapheresis (formally known as rheopheresis), which is our modification of double plasma filtration performed in the Hematological Department, University Hospital in Hradec Králové. Plasma is obtained not by filtration but by centrifugal separators. Blood is collected from a peripheral vein. Plasma is obtained by high-speed centrifugation (Cobe-Spectra or Optia blood cell separators, Terumo, Lakewood, Co, USA) and, in the second grade, is pumped through a high-molecular filter (Evaflux 4A, Kawasumi, Tokyo, Japan). This filter is made of ethylene-vinyl-alcohol hollow fibers with 0.03 mm sized holes, which captures a sizeable amount of LDL cholesterol, lipoprotein(a), fibrinogen, α _2_-macroglobulin and immunoglobulins (IgM in particular). After crossing the filter, plasma is returned, together with the formed elements of the blood, to the patient’s bloodstream. The filter is placed and controlled by the CF 100 instrument (Infomed, Geneva, Switzerland). In the case of increased pressure in the filter capillaries, the filter is automatically rinsed with a physiological solution, which is then discarded with the eliminated particles into the waste bag. The flow of plasma is continual; anticoagulation is ensured with heparin and ACD-A (Baxter, Munich, Germany); the amount of processed plasma: one and a half of body volume - is calculated by the blood cell separator computer. Duration of the procedure is approximately 2 h, which depends on influx of blood (status of peripheral veins). We prefer a peripheral venous access and only if they are insufficient do we use venous access via v. subclavia or v. femoralis. Contraindications of rheopheresis are identical to the general contraindications of hemapheresis - uncontrolled metabolic conditions (diabetes), cardiovascular disorders (unstable hypertension or coronary artery disease), malignancies, acute infections and cerebral insufficiency. Some other details have previously been described elsewhere [12, 15]. Patients with acute hearing loss underwent 3 procedures within one week.

Patients treated in the MicroWick arm were operated on in the standard conditions of an operating theater. With the patient in the supine position, the ear canal and eardrum of the impaired ear were cleaned of earwax and topically anesthetized using 10% lidocaine. On the cleaned ear canal and under microscopic control, the postero-inferior ear drum quadrant was incised by a sickle knife. The malleus handle was used as a landmark for localizing the round window membrane. A Silverstein MicroWick system was then smoothly inserted, and the wick was congested with dexamethasone solution (4 mg/mL). The patient was instructed to administer two drops of dexamethasone solution 4 times daily to the external ear canal of the hearing-impaired ear for a period of one month.

### Statistics

Descriptive statistics for demographic and baseline characteristics were summarized for all randomized patients. For treatment outcomes, the Fowler percentage of hearing loss on the affected ear was analyzed before and after treatment with the paired *T*-test or Wilcoxon test for non-parametric data. For comparison of independent groups, the *T*-test, non-parametric Mann—Whitney rank sum or one-way ANOVA test was used. For associations between factors, we used the Pearson Product Moment Correlation. Data are presented as mean (±SD) or median (lower, upper quartile). The significance level was set at *p* < 0.05. The statistical analyses were performed with SigmaPlot for Windows, version 11.0 (Systat Software, California, USA).

## Results

We examined 157 patients with SISHL, 110 of whom met the inclusion criteria, signed consent and entered into the study. One patient was rejected before the start of treatment for spontaneous remission. In the study group, 2 patients were lost to follow-up for personal reasons after completing corticosteroid-arm therapy and their final audiograms were missing. One patient was lost in the MicroWick arm for the same reason. The final number of 106 patients met the inclusion and exclusion criteria, were followed up for 12 months after treatment and enrolled in the statistical analysis.

**Fig. 1:**
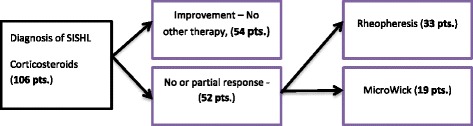
Study design

After initial corticosteroid therapy, 54 patients showed significant improvement - partial or complete recovery according to the guidelines [13] and required no other treatment (Standard arm). The rest - 52 patients chose either rheopheresis (33 patients) or MicroWick (19 patients) as shown in Fig. 1. The demographics and baseline audiological data of the patients are summarized in Table 1, which shows that there was no significant difference between the groups. Patients started steroid treatment after 4 days from onset of symptoms. Patients who failed steroid treatment entered rheopheresis after 9 days from onset symptoms, or the MicroWick arm after 10 days after onset of symptoms. There was no statistical difference in time to treatment between the MicroWick and rheopheresis arm (*p* = 0,279). Patients with more profound hearing loss were more likely to choose rheopheresis rather than MicroWick (81% (54, 98) vs. 52% (30, 92), *p* = 0.04) as is shown in Table 2.

**Table 1:** Demographic data

	Steroid therapy only (No. = 54)	Rheopheresis (No. = 33)	MicroWick (No. = 19)	
Age (median)	53 (38,65)	58 (44,66)	54 (29,61)	*p* = 0.234
Sex male/female	29/25	21/12	7/12	*p* = 0.175
Vertigo (%)	19	15	26	*p* = 0.609
Tinnitus (%)	74	79	74	*p* = 0.867
Time to treatment (days, median)	4 (1,7)	10 (8,15)	9 (6,14)	*p* <0.001
BMI (median)	25.7 (23,29)	26.6 (24,29)	24.1 (23,29)	*p* = 0.653

*No.* number of patients

**Table 2:** Comparison of hearing losses at time of follow-up (median in % of Fowler scale, (lower, upper quartile))

	Day 0/1 month		1 month/1 year	
Steroid therapy	54 (18,87)/13 (4, 50)	*p* < 0.001	13 (4,50)/11 (4, 29)	*p* = 0.224
Rheopheresis	81(54, 98)/54 (19, 74)	*p* < 0.001	54 (19, 74)/53 (25, 78)	*p* = 0.963
MicroWick	52 (30, 92)/52 (16, 83)	*p* = 0.940	52 (16, 83)/77 (14, 100)	*p* = 0.359

**Fig. 2:**
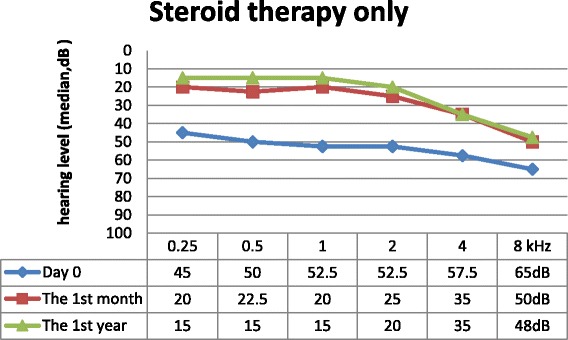
Results of standard therapy only

**Fig. 3:**
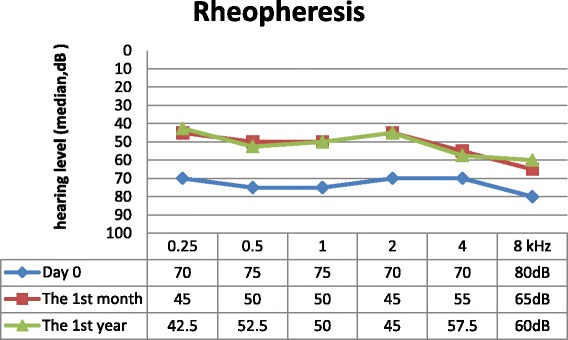
Results of rheopheretic arm

**Fig. 4:**
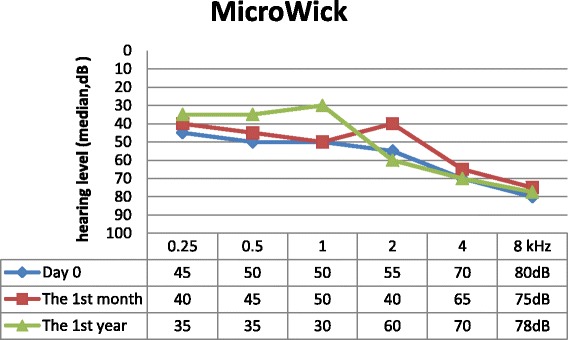
Results of MicroWick arm

We found age (*p* = 0.001), BMI (*p* = 0.0371) and vertigo (*p* = 0,041) to be positively correlated with the initial level of PTA. Sex (*p* = 0.285) and the presence of tinnitus (*p* = 0.567) were independent of initial hearing loss. Data from follow-up visits are shown in Figs. 2, 3 and 4. Hearing loss was significantly improved during the first month after the initial treatment in the steroid arm (*p* < 0.001) and in the rheopheresis arm (*p* < 0.001), and remained unchanged during the first year, as is shown in Table 2. Steroid-refractory patients in the MicroWick arm reached only mild, non-significant improvement within the first month (*p* = 0.940) after the treatment, and no further improvement was observed (*p* = 0.359). We observed a similar outcome in final absolute hearing loss levels in both arms after 1 month (*p* = 0.682). We found a positive correlation between final hearing loss and age (*p* = 0,003), and initial hearing loss (*p* < 0.001). Also patients with vertigo had higher final hearing loss (*p* = 0.006). Other factors showed no correlation with results (BMI (*p* = 0.23), tinnitus (*p* = 0.30) and sex (*p* = 0.878)).

There were 6 mild adverse events during steroid therapy: 4x decompensation of diabetes and 2x local complications after catheter insertion. Rheopheresis was complicated with 6 adverse events: 5x mild (short nausea during procedure) and 1x moderate (hypotension requiring saline infusion). In the MicroWick arm, we observed 7 adverse events: 2 mild – lasting perforation of tympanic membrane 1 year after procedure; in 3 other cases there was a need for operative occlusion (myringoplasty), classified as moderate according to CTCAE. In 2 other cases, there was progression to deafness on the treatment, which we classified as a serious adverse event.

## Discussion

Success of the treatment of any disorder depends on a full understanding of the underlying pathophysiological characteristics. Glucocorticoids exert a variety of immunosuppressive, anti-inflammatory, and anti-allergic effects on primary and secondary immune cells and tissues. Systemic steroid treatment is one of the few treatment options that has data showing efficacy and is recommended as first-line therapy in SHL according to 2012 guidelines [13, 16]. As a second-line treatment for non-responding patients (salvage therapy), several possibilities have been proposed: hyperbaric oxygen therapy, intratympanic steroid application, antiviral therapies, vasoactive agents, anticoagulants, rheopheresis and other [13]. Only some of these therapeutic options were proven effective in clinical trials.

We conducted a prospective, observational clinical trial of setting efficacy of rheopheresis and intratympanic steroid application (MicroWick system) in the treatment of systemic-steroid refractory SISHL. Efficacy of rheopheresis as the first-line therapy in comparison to corticosteroids was proven in other studies [7, 10, 17]. The superiority of rheopheresis over established first-line standard treatment could not be shown in general. Rheopheresis seems to be especially effective in patients with high fibrinogen or cholesterol [18]. Only few retrospective data are available [19] for refractory SISHL.

Efficacy of rheopheresis is based on the vascular theory of SISHL. Vascular compromise and associated cochlear ischemia are thought to be contributory to SISHL in some cases, or could be a final common pathway to hearing loss. And indeed atherosclerotic and rheological risk factors (hypercholesterolemia [20–22], hyperfibrinogenemia [23], age [24], BMI [22], metabolic syndrome [25] or hyperhomocysteinemia [26]) have recently been proven to be important in the etiology and prognosis of SISHL, although not all studies support such evidence [27, 28]. When analyzing our data, we cannot confirm an elevated baseline level of LDL-cholesterol, fibrinogen or viscosity in SISHL patients. However, after rheopheresis the level was significantly reduced in the case of some high molecular substances, such as cholesterol, immunoglobulin M, fibrinogen and others which have a significant influence on blood viscosity (Table 3.). This improves blood microcirculation and may be the main pathophysiological reason why rheopheresis is effective in the treatment of SISHL. During one procedure, the level of fibrinogen is reduced by 56%, similar to the other rheological important factors which we published elsewhere [12]. One procedure reduce total blood viscosity by 15.6%. We maintained reduced blood viscosity by repeating the procedures 3 times [12].

**Table 3:** Change in selected blood parameters (data available for 56 procedures)

	Before (± SD)	After(± SD)	p	Difference (%)
Fibrinogen (g/l)	3.22 (0.82)	1.43 (0.43)	<0.0001	−55.6
Plasma viscosity (mPa.sec)	2.11 (0.25)	1.82 (0.24)	<0.0001	−13.7
Blood viscosity (mPa.sec)	6.83 (1.6)	5.77 (1.07)	<0.0001	−15.6
Total cholesterol (mmol/L)	4.75 (1.01)	2.29 (0.54)	<0.0001	−51.8

As a secondary outcome, we searched for possible factors predicting steroid failure in the first-line therapy. All obtainable factors at the time of diagnosis (age, sex, vertigo, tinnitus, BMI,) failed to be predictive for steroid failure.

We used these factors for another analysis to learn whether these factors could serve as possible prognostic factors. Age (*p* = 0.003), vertigo (*p* = 0.006), and initial hearing loss (*p* < 0.001) were positively correlated with final outcome, while BMI (*p* = 0.23), tinnitus (*p* = 0.30) and sex (*p* = 0.878) were not. Other data (severity of hearing loss, time to treatment, BMI, vertigo) were identified as negative prognostic factors of SISHL in other trials [22–25]. When we performed the same analysis adjusted for therapeutic benefit (difference between initial and final hearing loss), only patients with higher initial hearing loss showed a significant positive correlation with hearing improvement (*p* < 0.001). The rest of the factors showed no prognostic relevance.

Safety evaluations included assessment of adverse events, clinically significant abnormal laboratory findings (i.e. blood chemistry, hematology, urinalysis), vital signs, and physical examination findings. Both procedures are safe, with more adverse events in the MicroWick arm. Two patients showed progression to deafness (evaluated as serious adverse events), which is a relatively high incidence. The cause was a long-lasting perforation of the eardrum - the prolonged open middle ear caused irritation of the middle ear and a series of complications (inflammatory and infectious) which could not be managed by therapy, so they progressed to complete deafness.

Our study has some important limitations. Out of the original 110 patients entering the study, 52 showed no improvement after steroid therapy and required other treatment. An optimal design with blind randomization into three arms (MicroWick, Rheopheresis and Placebo) was not possible due to the low number of patients and for ethical reasons (performing no therapy is impossible in patients requiring therapy). We could not even blindly randomize patients into two arms (patient’s refusal to undergo the invasive MicroWick system, contraindications for operation, patient’s wish, clinician’s opinion, etc.). From the results, it is evident that MicroWick and rheopheresis patients have different hearing losses - more severe cases of refractory SISHL were recruited into the rheopheretic arm (median loss 81% vs. 52%, *p* = 0.04, Table 2). In spite of the fact that such patients have significantly inferior prognoses, rheopheresis was able to significantly improve hearing loss to approximately the same level as was observed in the MicroWick arm. On the other hand, it is possible that in patients with more severe hearing loss, there is simply more room for recovery, which can explain the only mild-nonsignificant effect of the MicroWick system (*p* = 0.940, Table 2).

## Conclusions

The question of therapy for patients currently remains under discussion and a considerable number of patients are not improving spontaneously or following corticotherapy. Rheopheresis can be used as a potentially effective and safe salvage therapy for patients with steroid-refractory SISHL.
